# Flavonoids and Stilbenoids of the Genera *Dracaena* and *Sansevieria*: Structures and Bioactivities

**DOI:** 10.3390/molecules25112608

**Published:** 2020-06-03

**Authors:** Zaw Min Thu, Ko Ko Myo, Hnin Thanda Aung, Chabaco Armijos, Giovanni Vidari

**Affiliations:** 1 Department of Chemistry, Kalay University, Kalay 03044, Sagaing Region, Myanmar; kokomyokalay@gmail.com; 2 Department of Chemistry, University of Mandalay, Mandalay 100103, Myanmar; hninthandaaung07@gmail.com; 3 Departamento de Química y Ciencias Exactas, Universidad Técnica Particular de Loja, San Cayetano Alto s/n, Loja 1101608, Ecuador; 4 Medical Analysis Department, Faculty of Science, Tishk International University, Erbil 44001, Iraq

**Keywords:** *Dracaena*, *Sansevieria*, biological/pharmacological activities, flavonoids, stilbenoids

## Abstract

The genera *Dracaena* and *Sansevieria* (Asparagaceae, Nolinoideae) are still poorly resolved phylogenetically. Plants of these genera are commonly distributed in Africa, China, Southeast Asia, and America. Most of them are cultivated for ornamental and medicinal purposes and are used in various traditional medicines due to the wide range of ethnopharmacological properties. Extensive in vivo and in vitro tests have been carried out to prove the ethnopharmacological claims and other bioactivities. These investigations have been accompanied by the isolation and identification of hundreds of phytochemical constituents. The most characteristic metabolites are steroids, flavonoids, stilbenes, and saponins; many of them exhibit potent analgesic, anti-inflammatory, antimicrobial, antioxidant, antiproliferative, and cytotoxic activities. This review highlights the structures and bioactivities of flavonoids and stilbenoids isolated from *Dracaena* and *Sansevieria*.

## 1. Introduction

The taxonomic boundaries of the dracaenoid genera *Dracaena* and *Sansevieria* have long been debated. In the APG IV system of flowering plant classification, the two genera are still differentiated; both have been placed in the subfamily Nolinoideae of the family Asparagaceae, in the order Asparagales [1]. However, recent molecular phylogenetic studies showed that *Sansevieria* was nested within *Dracaena*, rendering the latter paraphyletic unless *Dracaena* was expanded to include species formerly placed in *Sansevieria* [2,3,4]. In this paper we have maintained the historical division in the two genera because the chemical literature is mainly based on the former botanical classification. However, known *Dracaena* synonyms for *Sansevieria* species are also reported.

The genus *Dracaena* consists of more than 100 accepted species which are mainly distributed in the tropics and subtropics, especially in Africa, Australia, and Southern Asia [5]. They are mainly succulent shrubs and trees, and a few are commonly grown as shrubby houseplants, especially the variegated forms. The complete chloroplast (CP) genomes of six species have recently been reported, showing that can be used as a super-barcode for *Dracaena* spp. identification [6].

**Figure 1:**
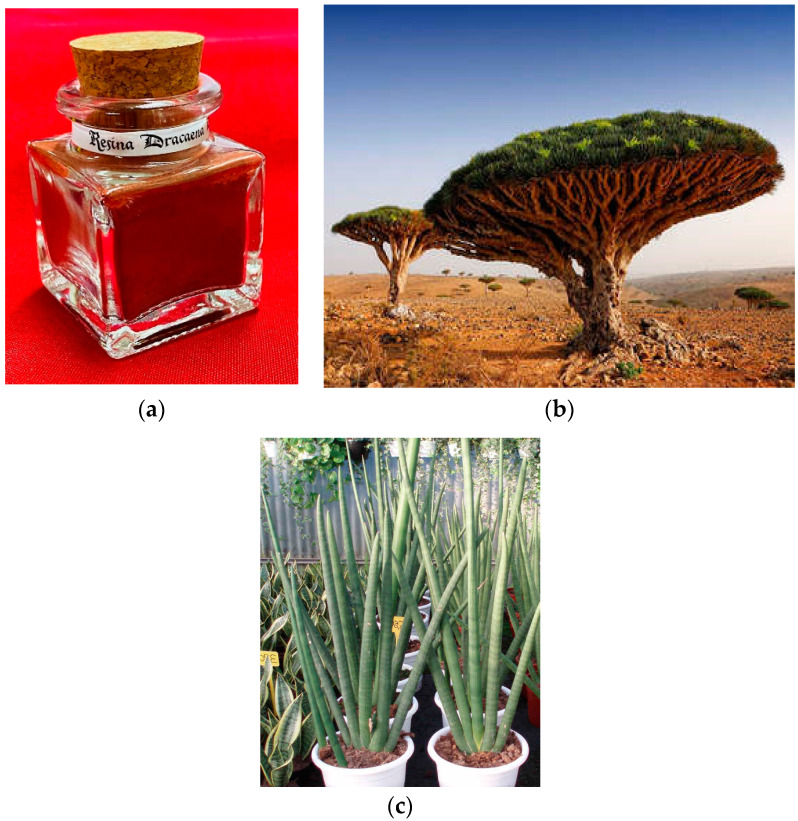
(**a**) Dragon’s blood; (**b**) *Dracaena cinnabari* Balf. f. (Socotra, Yemen); and (**c**) *Sansevieria cylindrica* Bojer ex Hook. The images have been downloaded from the Internet.

“Dragon’s blood” is a non-specific name for deep red resinous exudates that are obtained from cut stems of different plant taxa endemic to various regions around the globe and belonging to the families of Asparagaceae, Arecaceae, Chamaesyce (Euphorbiaceae), and Fabaceae [7,8]. About six *Dracaena* plants, growing in China, Southeast Asia, West Africa, Arabian Peninsula, Yemen, India, and Macaronesia are the main sources of this resin [6,8]. The drug (Figure 1a) was a commercially important export, notably from the island of Socotra (Yemen) (Figure 1b) and it has widely been used in traditional medicines through the world for thousands of years as an efficacious remedy for the treatment of hemorrhage, dysentery, diarrhea, stomach and external ulcers, wounds, leucorrhea, fractures, piles, diabetes, and even tumors [6,8,9,10,11]. In fact, the resin is known to have remarkable anti-inflammatory and antioxidant effects and to enhance immune function, promote skin repair, stop bleeding, and enhance blood circulation [8,9,10,12,13]. Dragon’s blood is included in the Pharmacopoeia of the People’s Republic of China, where it was first imported through the silk road during the Sui and Tang dynasties. Until the 1970s, the red resin of *Dracaena cochinchinensis* S. C. Chen, used originally by the Dai people, living in the south part of Chinese Yunnan province, for treating pain and stopping hemorrhages, was found and used as the substitute of the traditionally imported dragon’s blood, called Long-Xue-Jie (Chinese dragon’s blood) [9,10].

Besides medicinal uses, the resin has also been employed as a pigment in works of art since ancient times by many cultures. In addition to flavonoids and stilbenoids (*vide infra*), chemical and pharmacological studies have indicated that other phenolic compounds, saponins, and terpenes are the main effective compounds occurring in dragon’s blood [8,9,10,11].

The genus *Sansevieria* includes species (Figure 1c) with a distribution ranging from Africa, Madagascar through Asia to Myanmar and the islands of the Indian Ocean [14,15]. The genus comprises perennial herbs with erect, stiff leaves and short, thick, and stoloniferous rhizomes. Common names for the about 70 or more known species include mother-in-law’s tongue, devil’s tongue, cow tongue, bow-string hemp, snake plant, and zebra lily [14,15]. The genus was originally named *Sanseverinia* by Vincenzo Petagna in 1787, to honor his patron Pietro Antonio Sanseverino, Count of Chiaromonte (1724–1771), in whose garden Petagna saw the plant. In 1794 Thunberg used the name *Sansevieria* that, since that time, has been conserved in the botanical literature [16]. The name honors Italian scientist and inventor Raimondo di Sangro (1710–1771), Prince of Sanseviero. The spellings “*Sanseveria*” and “*Sanseviera*” are commonly used as well, the confusion deriving from alternate spellings of the Italian place name. The leaves and rhizomes of *Sansevieria* genus are used in folk medicine for treating asthma, cough, sexual weakness, hypertension, abdominal pains, colic, eczema, piles, edema, jaundice, anuria, palpitations, viral hepatitis, malaria, snake- and insect bites, etc. [15,17,18,19,20].

Besides the medicinal aspects, several *Dracaena* and *Sansevieria* species have great horticultural importance and are commercialized for use in landscaping and as indoor ornamental plants [21]. Moreover, it has been reported that *Dracaena* spp. can be used as bioindicators for the control of increasing air pollution in urban cities [22].

This review describes the most characteristic flavonoids and stilbenes isolated from *Dracaena* and *Sansevieria* species that have been investigated so far; the main biological activities are also reported. Compounds found in each species have been divided according to the plant organ from which they have been isolated. The literature has been retrieved from the databases Reaxys and Google Scholar until February 2020. A few papers written in Chinese (see reference [8]) could not be reviewed.

## 2. Bioactive Flavonoids and Stilbenoids from the Genera *Dracaena* and *Sansevieria*

### 2.1. Dracaena Species

#### 2.1.1. *Dracaena angustifolia* Medik, (Roxb.)

**Figure 2:**
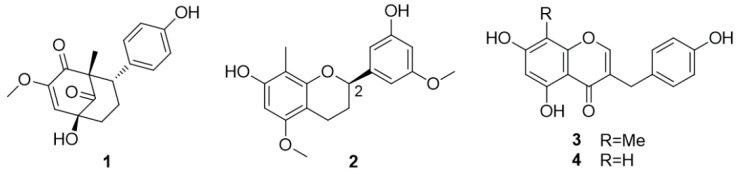
Chemical structures of compounds **1**–**4** isolated from *D. angustifolia.*

The native range of this shrub is from Bangladesh, through Indochina and Malaysia to Northern Australia. Tazettone H (structure **1** in Figure 2), (2*R*)-7,3′-dihydroxy-5′,5-dimethoxy-8-methylflavan (**2**), (2*R*)-7,4′-dihydroxy-5,3′-dimethoxy-8-methylflavone, (2*R*)-7,4′-dihydroxy-5-methoxy-8- methylflavanone, 5,4′-dihydroxy-7-methoxy-8-methylflavan, 4′-hydroxy-5,7-dimethoxy-8-methylflavan, (3*S*)-3,5,7-trihydroxy-4′-methoxyhomoisoflavanone, 5,7,4′-trihydroxy-6-methyl- homoisoflavanone, desmethylisoophiopogonone B (**3**), and 5,7,4′-trihydroxyhomoisoflavone (**4**) were isolated from the stems [23].

Compounds **2**–**4** displayed weak anti-inflammatory activity with IC_50_ values of 45, 33 and 61 μM, respectively [23].

#### 2.1.2. *Dracaena cambodiana* Pierre ex Gagnep

**Figure 3:**
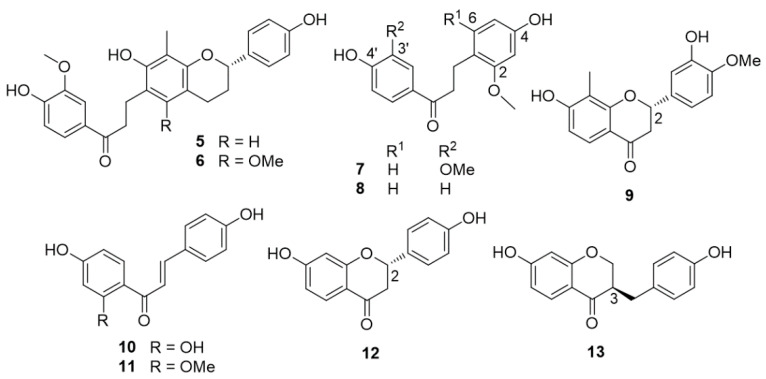
Chemical structures of compounds **5**–**13** isolated from *D. cambodiana.*

**Figure 4:**
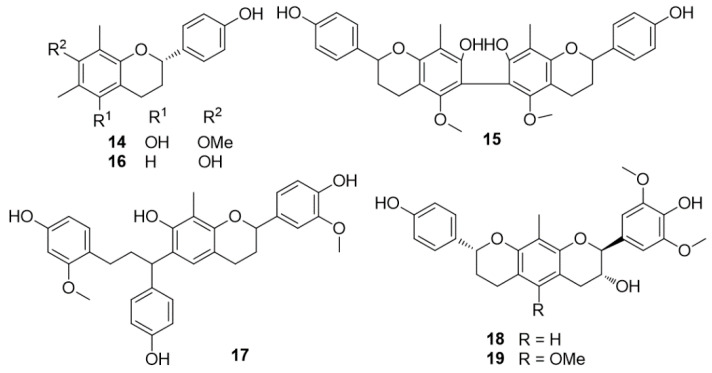
Chemical structures of compounds **14**–**19** isolated from *D. cambodiana.*

The plant grows in China and Southeast Asia. Intense investigations of dragon’s blood led to the isolation of cambodianins A (**5**) and B (**6**), 4,4′-dihydroxy-2,3′-dimethoxydihydrochalcone (**7**), 4,4′-dihydroxy-2-methoxydihydrochalcone (loureirin C) (**8**), 4,4′-dihydroxy-2,6-dimethoxy- dihydrochalcone, 4,2′,4′-trihydroxychalcone (**10**), 4,4′-dihydroxy-2′-methoxychalcone (**11**), (2*S*)-7,3′-dihydroxy-4′-methoxy-8-methylflavan (**9**), (2*S*)-7,4′-dihydroxyflavanone (**12**), (3*R*)-7,4′-dihydroxyhomoisoflavanone (**13**) (Figure 3), together with cambodianins D (**14**) and E (**15**), (2*S*)-7,4′-dihydroxy-6,8-dimethylflavan (**16**), cambodianins G (**18**) and H (**19**) (Figure 4) [24,25,26].

**Figure 5:**
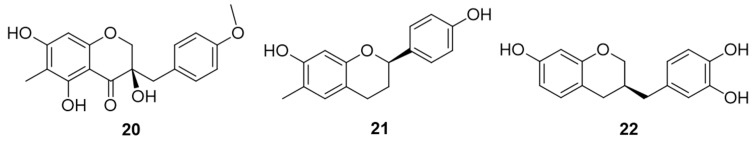
Chemical structures of compounds **20**–**22** isolated from *D. cambodiana.*

In an MTT assay, 4,4′-dihydroxy-2,6-dimethoxydihydrochalcone and compounds **5**, **6**, **11**, and **13** exhibited moderate cytotoxic effects against human myelogenous leukemia (K-562), human hepatocarcinoma (SMMC-7721), and human gastric tumor (SGC-7901) cell lines [24]. Compound **18** was moderately cytotoxic against K562 and SGC-7901 cell lines with IC_50_ values of 9.5 and 16.2 μg/mL, respectively [26]. Compounds **5**–**16, 18**, and **19** showed antibacterial activities against *Staphylococcus aureus* and compounds **5**, **6**, **7**, **8**, **10**, **12**, and **13**–**16** also exhibited antibacterial effects against methicillin-resistant *S. aureus* (MRSA) [24,25,26]. The dragon’s blood of *D. cambodiana* also produced 5,7-dihydroxy-4′-methoxy-8-methylflavan, 7,4′-dihydroxy-8-methoxyhomoisoflavan, (2*R*)-7,4′-dihydroxy-8-methylflavan, and (2*S*)-7,3′-dihydroxy-4′-methoxyflavan [27]. The Dai and Mei research group isolated the biflavonoids 8-methyl-3′-methoxysocotrin-4′-ol (cochinchinenin D) (structure **17** in Figure 4), 8-methyl-4′-methoxysocotrin-3′-ol, and 8-methylsocotrin-4′-ol, together with (2*S*)-7,4′-dihydroxy-6,8-dimethylflavan, (2*S*)-5,7-dihydroxy-4′-methoxy-8-methylflavan, (2*S*)-7,3′-dihydroxy-4′-methoxy-8-methylflavan (**9**), (2*R*)-7,4′-dihydroxy-8-methylflavan, (±)-7,3′-dihydroxy-4′-methoxyflavan, (±)-7,4′-dihydroxy-3′-methoxyflavan, and (2*S*)-7,4′-dihydroxyflavan from the stems of *D. cambodiana* [28,29,30]. The antimicrobial activities of these compounds were reported. In addition, a new homoisoflavonoid, named cambodianol (structure **20** in Figure 5), was isolated from the same sample.

Cambodianol exhibited high cytotoxic activity against chronic myelogenous leukemia (K562), human hepatoma (SMMC-7721), and human gastric cancer (SGC-7901) cells in an MTT assay with IC_50_ values of 1.4, 2.9, and 5.0 µg/mL, respectively, that were comparable with those of paclitaxel [30,31]. 7,4′-Dihydroxy-8-methoxyhomoisoflavan, 7,4′-dihydroxyhomoisoflavan, 4,4′-dihydroxy-2-methoxydihydrochalcone, 4,4′-dihydroxy-3,2′-dimethoxychalcone, 4,2′,4′-trihydroxychalcone, 4,4′-dihydroxy-2′-methoxychalcone were also isolated from *D. cambodiana* stems [32,33]. 4,4′-Dihydroxy-3,2′-dimethoxychalcone showed significant activity against chronic myelogenous leukemia (K562), human hepatoma (SMMC-7721), and human gastric cancer (SGC-7901) cells in an MTT assay, with IC_50_ values of 2.5, 4.3, and 4.4 mg/mL, respectively. These values were comparable with those of mitomycin C [32]. The antioxidant and radical scavenging activities of compounds **20**, (2*S*)-7,3′-dihydroxy-4′-methoxy-8-methylflavan (**9**), (2*R*)-7,4′-dihydroxy-8-methyl flavan, (±)-7,4′-dihydroxy-3′-methoxyflavan, (2*S*)-7,4′-dihydroxyflavan, 7,4′-dihydroxyhomoisoflavan, and 4,4′-dihydroxy-2′-methoxychalcone were from weak to moderate [33].

Dragon’s blood of *D. cambodiana*, artificially induced by mineral salt and acid injection into trunk of the plant by transfusion [31,34], produced a wide range of phenolic compounds, some of which were the same as those isolated from the natural red resin. They include (2*R*)-7,4′-dihydroxy-8-methylflavan, (2*R*)-7,4′-dihydroxy-6-methylflavan (**21**), (2*S*)-7,4′-dihydroxyflavan, (2*S*)-5,4′-dihydroxy-7-methoxy-6,8-dimethylflavan, (2*S*)-7,3′,4′-trihydroxy-8-methylflavan, (2*S*)-7,4′-dihydroxy-3′-methoxyflavan, (±)-7,4′-dihydroxy-3′-methoxyflavan, (2*S*)-5,7-dihydroxy-4′-methoxy-8-methylflavan, (2*S*)-7,3′-dihydroxy-4′-methoxy-8-methylflavan, (3*R*)-7,4′-dihydroxyhomoisoflavan, (3*R*)-7,4′-dihydroxy-8-methoxyhomoisoflavan, (3*R*)-7,3′,4′-trihydroxyhomoisoflavan (**22**) (Figure 5), 2,4,4′-trihydroxy-3′-methoxy-3-methydihydrochalcone, 2,4,4′-trihydroxy-3-methydihydrochalcone, 4,2′,4′-trihydroxy-3-methoxychalcone, (±)-5,7,4′-trihydroxy-6-methylhomoisoflavanone, (±)-7,4′-dihydroxyhomoisoflavanone, (3*S*)-7,3′-dihydroxy-4′-methoxyhomoisoflavanone, (3*R*)-cambodianol (**20**), (2*S*)-7,4′-dihydroxyflavanone, pterostilbene, 10-hydroxy-11-methoxydracaenone C, 10,11-dihydroxydracaenone C, (3*E*)-7-hydroxy-3-[(3-hydroxy-4-methoxybenzylidene)chroman-4-one, (3*E*)-7-hydroxy-3-(4-hydroxybenzylidene)chroman-4-one, 3′-methoxy-8-methylsocotrin-4′-ol, 4′-methoxy-8-methylsocotrin-3′-ol, and 8-methylsocotrin-4′-ol [31,34]. (±)-7,4′-Dihydroxyhomo- isoflavanone and 8-methylsocotrin-4′-ol exhibited moderate cytotoxic effects against human myeloid leukemia (K562) and human gastric tumor (SGC-7901) cell lines (IC_50_ = 16–29 μg/mL) [30], whereas compound **21** was weakly cytotoxic against human hepatoma (BEL-7402) cells (IC_50_ = 39.2 μM) [34]. Compound **22** showed significant acetylcholinesterase (AChE) inhibitory activity [34]. Most isolated compounds demonstrated antibacterial activity against *S. aureus*; instead, they were only weakly active against MRSA [31].

#### 2.1.3. *Dracaena cinnabari* Balf. f.

**Figure 6:**
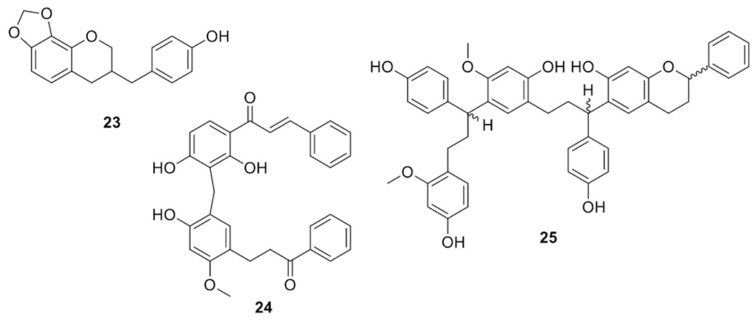
Chemical structures of compounds **23**–**25** isolated from *D. cinnabari.*

**Figure 7:**
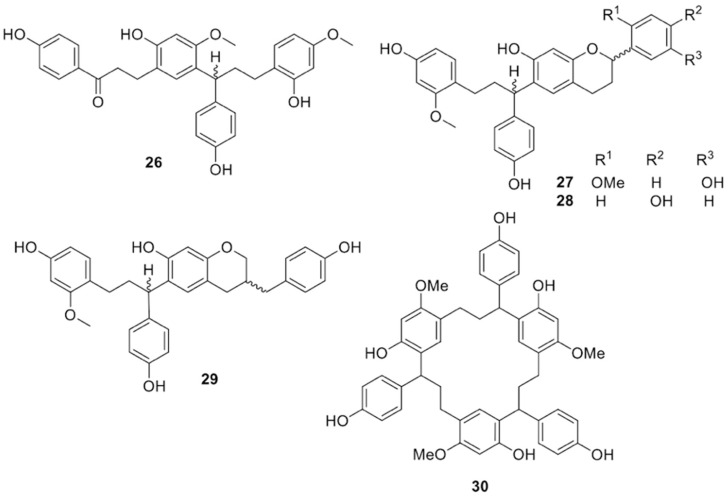
Chemical structures of compounds **26**–**30** isolated from *D. cinnabari.*

The plant grows in Arabian Peninsula and Yemen, especially in the island of Socotra (Figure 1b) where it has become a touristic attraction. From the red resin of the plant collected at Socotra, known in Arabic as Dammalakhawin or Cinnabar, Masaoud et al. isolated 7,3′-dihydroxy-4′-methoxyhomoisoflavan, 7,4′-dihydroxy-8-methoxyhomoisoflavan, 4′-hydroxy-7,8-methylendioxy- homoisoflavan (structure **23** in Figure 6), 7,4′-dihydroxyhomoisoflavan, (±)-7,4′-dihydroxy-3′-methoxyflavan, (2*S*)-7,3′-dihydroxy-4′-methoxyflavan, (2*S*)-7-hydroxyflavan, 4,4′-dihydroxy-2′-methoxychalcone, 4-hydroxy-2-methoxydihydrochalcone, 4,4′-dihydroxy-2-methoxydihydro- chalcone, (2*S*)-7-hydroxyflavanone, and 7,4′-dihydroxyflavone [35].

Compound **23** inhibited nitric oxide (NO), TNF-α, and IL-6 production in lipopolysaccharide- stimulated mouse macrophage RAW 264.7 cells [36], thus having potential anti-inflammatory activity.

Unprecedented phenols isolated from the resin were the C-linked chalcone-dihydrochalcone dimer dracidione (structure **24** in Figure 6) [37], the triflavonoid damalachawin (structure **25** in Figure 6) [38], the biflavonoids cinnabarone (**26**) [39], 2′-methoxysocotrin-5′-ol (**27**), socotrin-4′-ol (**28**), and homoisosocotrin-4′-ol (**29**) [40], and the metacyclophane dracophane (structure **30** in Figure 7) [41]. Dracidione (**24**) showed moderate α-glucosidase inhibitory activity (IC_50_ = 40.27 µg/mL) [37].

#### 2.1.4. *Dracaena cochinchinensis* (Lour.) S.C. Chen

**Figure 8:**
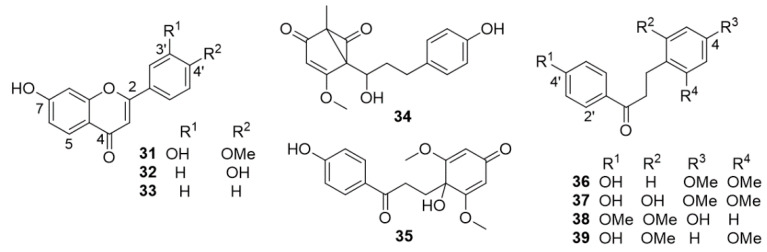
Selected flavones and dihydrochalcone derivatives from *D. cochinchinensis* red resin.

**Figure 9:**
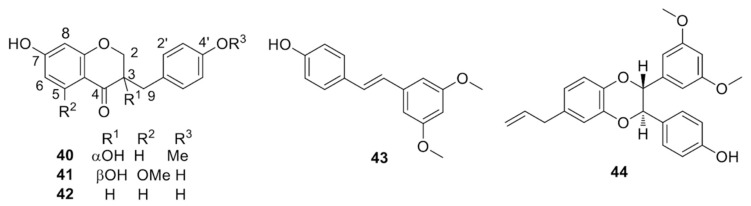
Selected homoisoflavanones and stilbenoids from *D. cochinchinensis* red resin.

**Figure 10:**
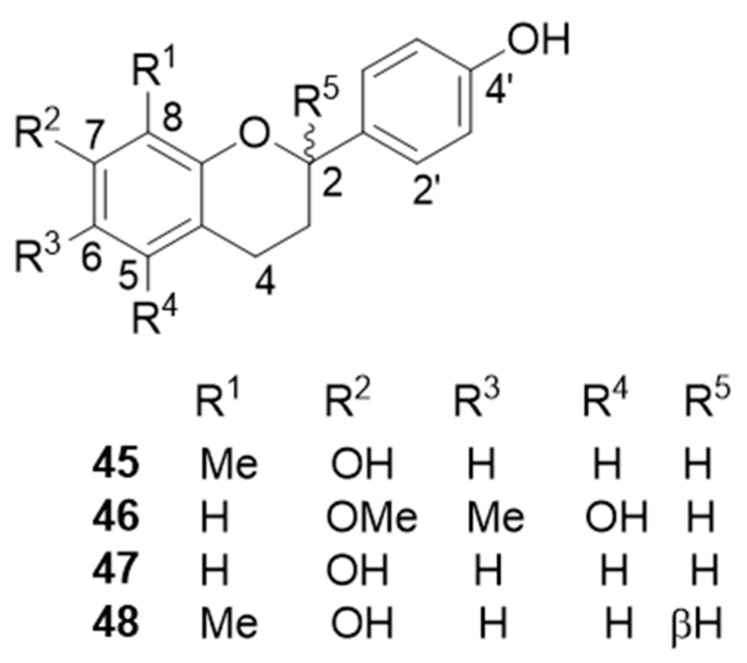
Selected flavans from *D. cochinchinensis* red resin.

**Figure 11:**
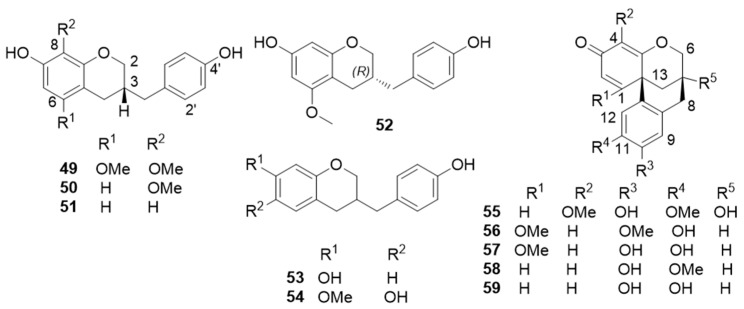
Selected homoisoflavans and meta-homoisoflavans from *D. cochinchinensis* red resin.

**Figure 12:**
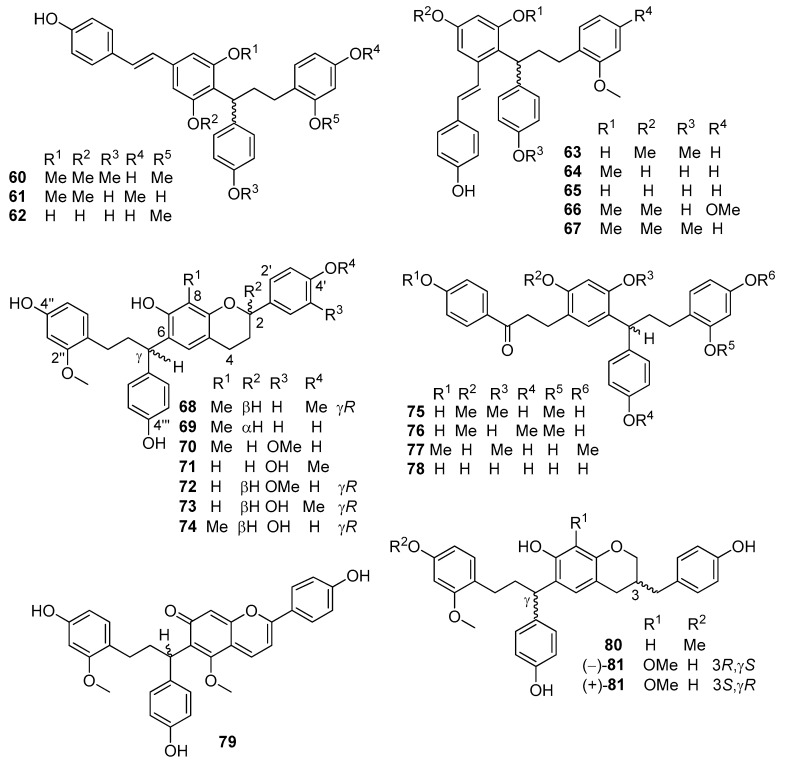
Selected flavonoid dimers from *D. cochinchinensis* red resin.

**Figure 13:**
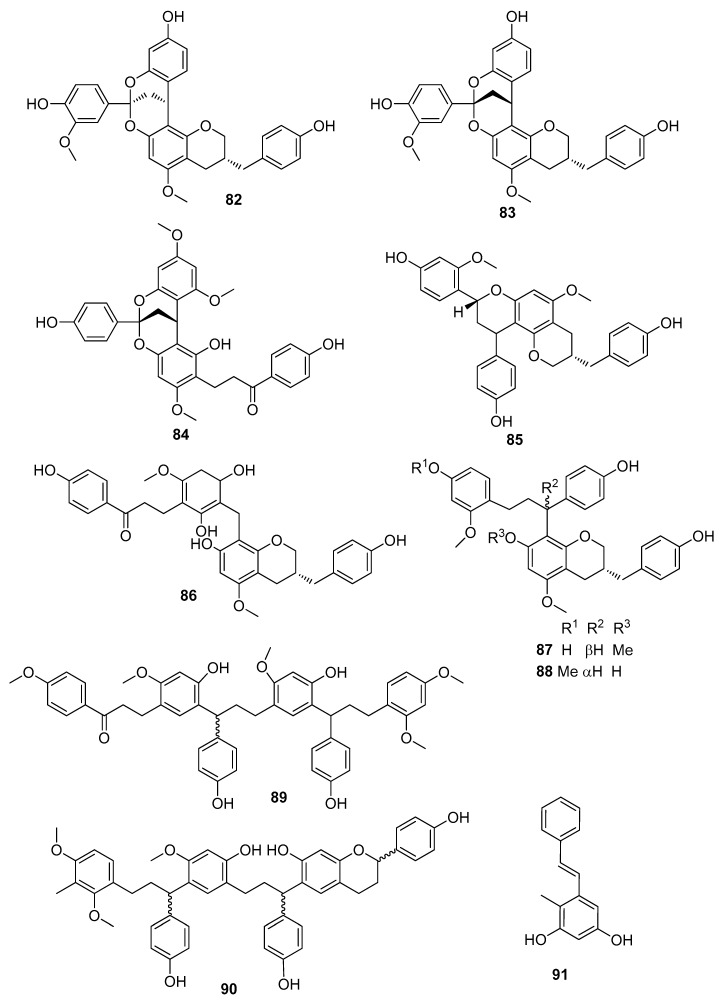
Selected flavonoid dimers (*cont*.) and trimers from *D. cochinchinensis* red resin.

**Figure 14:**
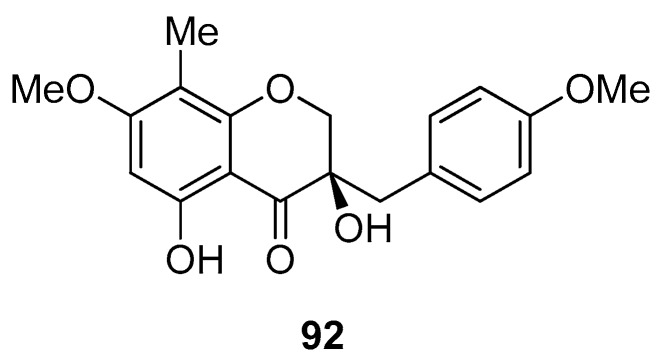
Chemical structure of dracol isolated from *D. draco.*

**Figure 15:**
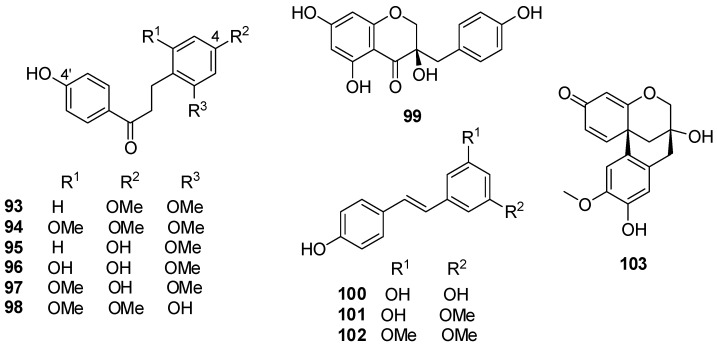
Chemical structures of compounds **93**–**103** isolated from *D. loureiri.*

**Figure 16:**
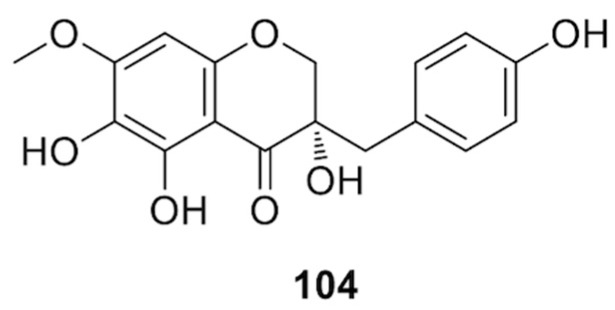
Chemical structure of compound **104** isolated from *D. usambarensis.*

The plant grows in China and Southeast Asia. Phytochemical and pharmacological investigations performed on *D. cochinchinensis* extracts are innumerable, given the wide use of the drug in the traditional Chinese medicine. Several extracts of the plant, especially of the red resin, known as Chinese dragon’s blood or Yunnan dragon’s blood, have been true mines of a great number of novel compounds with intriguing structures (Figure 8, Figure 9, Figure 10, Figure 11, Figure 12, Figure 13, Figure 14, Figure 15 and Figure 16). The immunomodulatory, antibacterial, antiviral, antidiarrheal, antioxidant, antiatherosclerosis, antiulcer, antiseptic, mutagenic and antimutagenic, antitumor, anticancer, and cytotoxic effects of Chinese dragon’s blood have been proved by pharmacological investigations [42]. The drug is commonly prescribed to improve blood circulation, to stop hemorrhages, and to heal wound cuts and pains [42]. It has also been used to treat a diverse range of peripheral inflammation and central inflammation-associated diseases such as diabetes, arthritis, colitis, gynecopathy, and allergic dermatitis [42]. A Chinese pharmaceutical formulation, named Longxuetongluo Capsule (LTC), which is derived from the total phenolic extract of Chinese dragon’s blood, has been proved to be safe as well as effective against ischemic stroke. At least ten different research groups, mainly from China, have isolated and determined the structures of a great number of flavonoids and stilbenoids from the red resin. To avoid repetitions, compounds have been grouped according to their biosynthetic families. Flavones (Figure 8): 7-hydroxy-6-methoxyflavone [43], 7,3′-dihydroxy-4′-methoxyflavone (**31**) [44], 7,4′-dihydroxyflavone (**32**) [44,45,46], 7-hydroxyflavone (**33**) [42], 5,7,4′-trihydroxy-8-methylflavone [42], 5,7,4′-trihydroxyflavone (apigenin) [42]. Flavanones*:* 7,4′-dihydroxyflavanone (liquiritigenin) [42,44], (2*S*)-5,7-dihydroxyflavanone (pinocembrin) [42]. Chalcones*:* 4,4′-dihydroxy-2-methoxychalcone (echinatin) [42,46], 4,4′-dihydroxy-3′-methoxychalcone [44], 4,4′-dihydroxy-2′-methoxychalcone (**11**) [44,47]. Dihydrochalcones derivatives (Figure 8): 6-methoxy-3-methyl-(1-hydroxy-3-(4-hydroxyphenyl)propyl)-bicyclo[3.1.0]hex-6-ene-2,4-dione (“cochinchinenene H”) (**34**) [42], 4-hydroxy-2,4′-dimethoxy- dihydrochalcone [48], 3,4′-dihydroxy-2,4,6-trimethoxydihydrochalcone [48], 2,4,4′-trihydroxy- dihydrochalcone [47,48], cochinchinenone (**35**) [47], 4′-hydroxy-2,4-dimethoxydihydrochalcone (loureirin A) (**36**) [43,45,46,47,48], 4′-hydroxy-2,4,6-trimethoxydihydrochalcone (loureirin B) [43,45,46,48], 2,4′-dihydroxy-4,6-dimethoxydihydrochalcone (**37**) [48], loureirin C (**8**) [42,44,46,47,48], 4,4′-dihydroxy-2,6-dimethoxydihydrochalcone [42,44,46,47,49], 4,6,4′-trihydroxy-2-methoxydihydrochalcone [47], 4′-hydroxy-4,2′-dimethoxydihydrochalcone (**38**) [50], and 4-hydroxy-2′,6′- dimethoxydihydro-chalcone (**39**) [51].

Homoisoflavones*:* 5,4′-dihydroxy-7-methoxyhomoisoflavone [52], 7,4′-dihydroxyhomoisoflavone [46], 7-hydroxy-3-(4-hydroxy-benzylidene)chroman-4-one [42]. Homoisoflavanones (Figure 9): (3S)-3,7-dihydroxy-4′-methoxyhomoisoflavanone [(3*S*)-dracaeconolide A] (**40**) [49], (3*S*)-3,7,4′-trihydroxy-5-methoxyhomoisoflavanone (**41**), (3*S*)-7,4′-dihydroxy-5-methoxyhomoisoflavanone [42,52], 7,4′-dihydroxyhomoisoflavanone (**42**) [42,43,52], (3*S*)-7,4′-dihydroxyhomoisoflavanone [44], 3,5,7,4′-tetrahydroxyhomoisoflavanone (loureiriol) [52], and 7,4′-dihydroxy-8-methoxyhomoisoflavanone [47]. Stilbenoids (Figure 9): *trans*-resveratrol [42,44], 3,4′-dihydroxy-5-methoxystilbene [42], *cis*-resveratrol [42], *trans*-3,5-dihydroxy-4′-methoxystilbene [47], *trans*-3,5,4′-trihydroxystilbene [47], pterostilbene (4′-hydroxy-3,5-dimethoxystilbene) (**43**) [43], 3-methylresveratrol [44], and cochinchin (structure **44** in Figure 9) [45].

Flavans (Figure 10): 7,4′-dihydroxy-8-methylflavan (**45**) [43,44,49], 4,4′-dihydroxy-2,6-dimethoxydihydrochalcone [47,48], (2*R*)-7,4′-dihydroxy-5-methoxy-8-methylflavan [49], 5,4′-dihydroxy-7-methoxy-6-methylflavan (**46**) [44,46,49], 7,4′-dihydroxy-3′-methoxyflavan [49], 7,4′-dihydroxyflavan (**47**) [46,49], (–)-7-hydroxy-4′-methoxyflavan [43,45,46,47], (2*S*)-7,4′-dihydroxy-8-methylflavan (**48**) [46,47], (2*S*)-5,4′-dihydroxy-7-methoxy-8-methylflavan [47], (2*R*)-4′-hydroxy-7-methoxy-8-methylflavan [47], and 6,4′-dihydroxy-7-methoxy-8-methylflavan [46].

Homoisoflavans and meta-homoisoflavans (Figure 11)*:* (3*R*)-7,4′-dihydroxy-5,8-dimethoxy- homoisoflavan [(3*R*)-dracaeconolide B] (**49**) [49] (Figure 11) (3*R*)-7,4′-dihydroxy-8-methoxy- homoisoflavan (**50**) [48,49,53], (3*R*)-7,4′-dihydroxyhomoisoflavan (**51**) [45,46,48,49], (3*R*)-7,4′-dihydroxy-5-methoxyhomoisoflavan (**52**) [42,46,48,49], 7,4′-dihydroxyhomoisoflavan (**53**) [43,44], (3*R*)-7,4′-dihydroxy-6-methoxyhomoisoflavan [52], 6,4′-dihydroxy-7-methoxyhomoisoflavan (**54**) [43,46], (3*R*)-6,4′-dihydroxy-8-methoxyhomoisoflavan [49], 7,3′-dihydroxy-8,4′-dimethoxyhomo- isoflavan [48], 4′-hydroxy-7,8-dimethoxyhomoisoflavan [48], 4′-hydroxy-5,7-dimethoxyhomo- isoflavan [48], (7*R*,12b*R*)-7,10-dihydroxy-4,11-dimethoxydracaenone (**55**) [52], (7*S*,12b*S*)-11-hydroxy-1,10-dimethoxydracaenone (**56**) [52], (7*S*,12b*S*)-10,11-dihydroxy-1-methoxydracaenone (**57**) [52], 10-hydroxy-11-methoxydracaenone (**58**) [52], 10,11-dihydroxydracaenone C (**59**) [44], and 4′-hydroxy-7,8-methylenedioxyhomoisoflavan (**23**) [46,48].

Flavonoid dimers and trimers (Figure 12 and Figure 13): cochinchinenenes A–D (structures **60**,**63**–**65** in Figure 11) [44,47,53], cochinchinenenes E (**66**) and F (**61**) [43], cochinchinenenes G (**67**) and H (**62**) [44], “cochinchinenene G” (**68**) [42], (2*R*)-8-methylsocotrin-4′-ol (**69**) [47], cochinchinenin [44], cochinchinenin B (**75**) [47], (γ*S*)-1-[5-(4,4′-dihydroxy-2-methoxydihydrochalconyl)]-1-(4-hydroxyphenyl)-3-(2-methoxy-4-hydroxy-phenyl)propane (cochinchinenin C) (**76**) [42,47], 1-[5-(2-methoxy-4,4′-dihydroxydihydrochalconyl)]-1-(4-hydroxyphenyl)-3-(2-methoxy-4-hydroxy-phenyl)- propane [44,47], cochinchinenins D-H (structures **70**,**80**,**77**,**90**,**89** in Figure 12 and Figure 13), cinnabarone (**26**) [39], dracaenin A (**79**) [42,54], socotrin-4′-ol (**28**) and homoisosocotrin-4′-ol (**29**)[40], cochinchinenins I-M (structures **78**,**81**,**71**,**72**,**73** in Figure 12), (2*RγS*)-3′-methoxy-8-methylsocotrin-4′-ol, (2*SγR*)-3′-methoxy-8-methylsocotrin-4′-ol, (2*RγR*)-8-methylsocotrin-4′-ol, (2*RγS*)-8-methyl- socotrin-4′-ol [55,56,57], compound **74** [53], and biflavocochins A-G (structures **82**–**88** in Figure 13) [53].

It is worth noting that the names cochinchinenenes G and H, at first attributed to compounds **67** and **62**, respectively [44], were later also used for naming “compounds” **68** and **34**, respectively [42].

(7*E*)-1,5-Dihydroxy-11,12,13-trimethoxystilbene, (7*E*)-1,5-dihydroxy-11,13-dimethoxystilbene, (7*E*)-2,4-dihydroxy-1-methylstilbene (structure **91** in Figure 13), 1,3-dihydroxy-2,4-dimethyl-5-methoxychalcone, (2*S*)-7,4′-dimethoxy-5-hydroxy-6-methylflavan, and (3*S*)-3,7-dihydroxy-4-methoxyhomoisoflavanone were identified by Niu and his co-workers in dragon’s blood of *D. cochinchinensis* elicited by *Fusarium graminearum* [58]. Flavonoids and stilbenoids contained in other parts of the plant have been less investigated than the flavonoids isolated from the resin. (2*S*)-4′,7-dihydroxy-3′-methoxy-8-methylflavan, (2*S*)-7,3′-dihydroxy-4′-methoxy-8-methylflavan (**9**), 7-hydroxy-4′-methoxyhomoisoflavanone, (3*R*)-3,7-dihydroxy-8,4′-dimethoxyhomoisoflavanone, and 4,2′,4′-trihydroxychalcone were isolated from *D. cochinchinensis* leaves [59]. 7,4′-Dihydroxy- homoisoflavanone (**42**), 10,11-dihydroxydracaenone C (**59**), 7,4′-dihydroxyflavone, 7,4′-dihydroxy- flavan, 4,4′-dihydroxy-2-methoxychalcone (echinatin), 4,4′-dihydroxy-2-methoxydihydrochalcone, and 7,4′-homoisoflavan, together with sterols and phenolic glycosides, were isolated from fresh stems [60].

Compounds **49**–**51** and **45**–**47** did not show cytotoxic effects at a concentration of 10 µM, whereas they significantly promoted osteogenic differentiation of mesenchymal stem cells (MSCs) by increasing the levels of alkaline phosphatase (ALP) activity to 159.6 ± 5.9%, 167.6 ± 10.9%, 162.0 ± 1.4%, 151.3 ± 4.0%, 171.0 ± 8.2%, and 169.9 ± 7.3%, respectively, relative to the control [49]. Compounds **72** and **73** showed significant inhibitory activities against NO production in lipopolysaccharide-stimulated BV-2 microglial cells with IC_50_ values of 4.9 ± 0.4 and 5.4 ± 0.6 μM, respectively [56], whereas IC_50_ values of compounds **41**, **42**, and **58** were in the range of 60.4–75.6 μM [52]. Apigenin, 7-hydroxy-3-(4-hydroxybenzylidene)chroman-4-one, compounds **8**, **31**–**33**, **52**, and **68** exhibited significant anti-neuroinflammatory properties without showing cytotoxic effects at the tested concentrations. A primary mechanistic study revealed that these effective compounds could inhibit neuroinflammation by inhibiting NO production and reducing the expressions of iNOS, IL-6, and TNF-α in LPS-activated BV2 microglial cells [42]. Compounds **60**, **63**–**65**, **69**, **75**, **76**, and 1-[5-(2-methoxy-4,4′-dihydroxydihydrochalconyl)]-1-(4-hydroxyphenyl)-3-(2-methoxy-4-hydroxyphenyl)- propane showed moderate thrombin inhibitory activity, whereas compounds **48**, **75**, and **76** were active against *Helicobacter pylori* (ATCC43504) with MIC values of 29.5, 29.5, and 31.3 μM, respectively [47]. Compounds **43**, **58**, **61**, and **66** showed good NAD(P)H Quinone Dehydrogenase 1 (NQO1) inducing activities, whereas compounds **36**, **37**, **43**, **46**, **53**, and **54** exhibited anti-inflammatory activities [43]. Compound **82** showed neuroprotective effect on serum deficiency-induced cellular damage in neuroendocrine PC12 cells (pheochromocytoma cells derived from the adrenal gland of *Rattus norvegicus*), whereas compounds **83**, **87**, and **88** exhibited moderate protein tyrosine phosphatase 1B (PTP1B) inhibitory activities [53]. Compound **91** exhibited potent antioxidant activity and a broad-spectrum of inhibitory activity against fungal strains, including *Exserohilum turcicum*, *Bipolaris maydis*, *Curvularia lunata,* and *F. graminearum* [58].

#### 2.1.5. *Dracaena draco* L.

*D. draco* L. is native to India, the Canary Islands, Cape Verde, Madeira, and locally in Western Morocco. It is the natural symbol of Tenerife island. Independently, an Italian research group [61] and Gonzàlez and collaborators [62] isolated from the red resin of *D. draco* the following group of compounds: (2*S*)-7,4′-dihydroxy-3′-methoxy-8-methylflavan, (2*S*)-5,4′-dihydroxy-7-methoxy-8-methylflavan, (2*S*)-7,4′-dihydroxy-3′-methoxyflavan, (2*S*)-7,4′-dihydroxy-8-methylflavan, (2*S*)-7,3′-dihydroxy-4′-methoxy-8-methylflavan (**9**), 7,4′-dihydroxyhomoisoflavan, 7,4′-dihydroxy-8-methoxyhomoisoflavan, 4′-hydroxy-5,7-dimethoxyhomoisoflavan, 4′-hydroxy-7,8-methylendioxy- homoisoflavan, 5,7,4′-trihydroxyhomoisoflavan, 10-hydroxy-11-methoxydracaenone (**42**), 3,4′-dihydroxy-7-methoxyflavone, liquiritigenin (7,4′-dihydroxyflavanone), 7,4′-dihydroxyhomoiso- flavanone, 5,7,4′-trihydroxy-6-methylhomoisoflavanone, 5,7,4′-trihydroxyhomoisoflavanone, 4,4′-dihydroxy-2′-methoxychalcone, isoliquiritigenin (4,2′,4′-trihydroxychalcone), 2,4,4′-trihydroxy- dihydrochalcone, and loureirin C (4,4′-dihydroxy-2-methoxydihydrochalcone) (**8**). Flavans of dragon’s blood from *D. draco* L. *subsp. draco* (Tenerife and Cape Verde) and *D. draco* L. *subsp. ajgal* (Morocco) were identified as (2*S*)-7,3′-dihydroxy-4′-methoxy-8-methylflavan (**9**), (2*S*)-7,4′-dihydroxy-3′-methoxy-8-methylflavan, (2*S*)-7,4′-dihydroxy-3′-methoxyflavan, (2*S*)-7,4′-dihydroxy-8-methylflavan, and (2*S*)-5,4′-dihydroxy-7-methoxy-8-methylflavan [63]. From samples of dragon’s blood collected from ancients specimens of *D. draco* L. *subsp. draco* growing in Palermo, Di Stefano reported the isolation and structural characterization of 7,4′-dihydroxyflavan, 7,3′-dihydroxy- 4′-methoxy-8-methylflavan, 7,4′-dihydroxy-3′-methoxy-8-methylflavan, 7,4′-dihydroxy-3′-methoxy- flavan, 7,4′-dihydroxy-8-methylflavan, 5,4′-dihydroxy-7-methoxy-8-methylflavan, 7,4′-dihydroxy- homoisoflavan, 7,4′-dihydroxy-8-methylhomoisoflavan, 7,4′-dihydroxy-8-methoxyhomoisoflavan, 7,4′-dihydroxy-5-methoxyhomoisoflavan, 7,4′-dihydroxyhomoisoflavanone, 5,7,4′-trihydroxyhomo- isoflavanone, 5,8,4′-trihydroxy-7-methoxyhomoisoflavanone, 5,7,4′-trihydroxy-6-methoxyhomo- isoflavanone, 5,7,4′-trihydroxy-6-methylhomoisoflavanone, and 5,7,4′-trihydroxy-6,8-dimethyl- homoisoflavanone [64]. Dracol (structure **92** in Figure 14), 5,7,4′-trihydroxyhomoisoflavanone, and helichrysetin were isolated from an EtOH extract of *D. draco* leaves [65]. Compound **92** was completely ineffective against HL-60 (leukemia), A431 (epidermoid), HeLa (cervix), and SK-OV-3 (ovarian) tumor cells (IC_50_ values > 100 µM) [65]. Quercetin-3-*O*-rutinoside was identified in aqueous extract of *D. draco* fruits [13]. 4′-Hydroxy-7,8-methylendioxyhomoisoflavan, (2*S*)-7,4′-dihydroxy-8-methylflavan, 7,4′-dihydroxy-8-methoxyhomoisoflavan, loureirins A and C, (-)-7,3′-dihydroxy-4′-methoxy-8-methylflavan, 7,4′-dihydroxyhomoisoflavan, isoliquiritigenin. (2*S*)-7,4′-dihydroxy-3′-methoxyflavan, and (2*S*)-5,4′-dihydroxy-7-methoxy-8-methylflavan were isolated from *D. draco* roots [65]. Loureirin C (**8**), (2*S*)-7,4′-dihydroxy-3′-methoxyflavan, and isoliquiritigenin [66,67] were isolated from *D. draco* stem bark [67].

#### 2.1.6. *Dracaena loureiri* Gagnep

*D. loureiri* Gagnep is a misspelled name for *D. loureiroi* Gagnep, which is considered illegitimate and synonym for *Dracaena cochinchinensis* (Lour.) S.C. Chen [68]. The plant is known by Thai’s as ‘Jun-Par’ and is commonly referred to as “Thai Dracaena”. This herb has positive activities of promoting blood circulation for removing blood stasis, regenerating tissue to heal wound, relieving pain, and eliminating swelling. It has been commonly used for the treatment of coronary heart diseases, angina, and acute myocardial infarction. Moreover, the EtOAc extract of *D. loureiri* can promote inflammatory response induced by LPS through inhibiting ROS production in vascular smooth muscle cells and macrophages. Meksuriyen and Cordell isolated loureirins A–D (structures **93**–**96** in Figure 15) from the leaves of the plant collected in Thailand [69]. Loureirin D (**96**) exhibited mild inhibition of LPS-stimulated NO production in RAW 264.7 macrophages, with an IC_50_ value of 50.3 μM, whereas it inhibited the activation of the IL-6/STAT3/NF-κB signaling pathway [48,70]. Ichikawa et al. isolated loureirins B (**94**) and D (**96**), 4,4′-dihydroxy-2,6-dimethoxydihydrochalcone (**97**), 2,4′-dihydroxy-4,6-dimethoxydihydrochalcone (**98**), 3,5,7-trihydroxy-4′-methoxyhomoisoflavanone (eucomol), 5,7,4′-trihydroxyhomoisoflavanone, and 7,4′-dihydroxy-5-methoxyhomoisoflavanone from the stem wood of the plant, a Thai folkloric medicine called “Chan-daeng” [71]. Likhitwitayawuid also isolated loureiriol (**99**), 4,6,4′-trihydroxy-2-methoxydihydrochalcone, 4,3′,5′-trihydroxystilbene (**100**), 4,3′-dihydroxy-5′-methoxystilbene (**101**), and 4-hydroxy-3′,5′-dimethoxy- stilbene (**102**) [72]. Compounds **97** and **98** were estrogen agonist and stimulated the estrogen-dependent human breast adenocarcinoma MCF-7 cell proliferation in a concentration-dependent manner between 10^−8^ and 10^−5^ M. 5,7,4′-Trihydroxyhomoisoflavanone showed appreciable estrogenic activity, competing with [^3^*H*]-estradiol for binding to the bovine uterine estrogen receptor. The apparent IC_50_ value was 375 nM, compared to the value of 225 nM for genistein [71]. Compounds **100**–**102** showed potent inhibitory activity of COX-1 and COX-2 with IC_50_ values in the range between 1.29 and 4.92 µM [72].

The bioactivities of loureirin B (**94**) are worthy of note. The compound can modulate the TTX-R sodium channel in DRG neurons via an AC/cAMP/PKA pathway that involves the activation of AC and PKA [73]. Another study confirmed that compound **94** promotes insulin secretion and lowers blood glucose level mainly through increasing mRNA level of *Pdx-1*, *MafA*, and intracellular ATP level, and inhibiting K_ATP_ current and influx of intracellular Ca^2+^ [74]. Experiments on Crohn’s disease (CD) rat model induced by 2,4,6-trinitrobenzenesulfonic acid (TNBS) indicated that loureirin B (**94**) can be beneficial for ameliorating the damage to colon length in a dose dependent manner. Moreover, loureirin B remarkably ameliorated TNBS-induced inflammatory response via regulation of cytokines in the colonic tissues, and inhibited apoptosis through regulation of IL-6/STAT3/NF-κB signaling pathway. This finding may represent a novel approach to treat CD and provides an alternative choice for disorders associated with CD [70]. Homoisoflavans **69** and 7,10-dihydroxy-11-methoxydracaenone (structure **103** in Figure 15) were isolated from a CHCl_3_ extract of the plant. They were inactive against *S. aureus*, *Bacillus subtilis*, and *Escherichia coli* (MIC > 250 µg/mL) [75].

#### 2.1.7. *Dracaena usambarensis* Engl. (synonym for *Dracaena mannii* Baker)

The plant is distributed from Senegal to Angola along the African west coast; it is widespread in tropical Africa and grows along the African east coast from Kenya to Kosi Bay in northern KwaZulu-Natal. Separation of a root extract afforded 4,4′-dihydroxy-2,3-dimethoxydihydrochalcone, the homoisoflavonoid 7-*O*-methyl-8-demethoxy-3-hydroxy-3,9-dihydropunctatin, and the homoisoflavanones **99** and (3*S*)-3,5,6,4′-tetrahydroxy-7-methoxyhomoisoflavanone (structure **104** in Figure 16) [76]. Compound **104** showed moderate cytotoxic effects against drug sensitive human lymphoblastic leukemia CCRF-CEM T cells; instead, it was inactive against all the other tested cell lines, including leukemia (CEM/ADR5000), human breast adenocarcinoma (MDA-MB-231-pcDNA3 and MDA-MB-231-BCRP clone 23), human glioblastoma (U87.MG and the resistant subline U87.MGΔEGFR), human hepatocyte carcinoma (Hep G2), and healthy hepatocyte (AML12) cell lines [76].

#### 2.1.8. Other *Dracaena* spp.

(2S)-7,4′-Dihydroxy-8-methylflavan and (2S)-5,4′-dihydroxy-7-methoxy-8-methylflavan were isolated from the dragon’s blood of *Dracaena tamaranae* Marrero Rodr., R. S. Almeira, and M. Gonzales-Martin (Gran Canaria) [63]. The common flavonoids rutin, kaempferol, and quercetin were isolated from a methanolic extract of the leaves of *Dracaena ombet* Heuglin ex Kotschy and Peyr. [77]. Kaempferol 3-*O*-α-l-rhamnopyranosyl-(1→2)-*O*-[α-l-rhamnopyranosyl-(1→6)]-β-d-galacto- pyranoside and kaempferol 3-*O*-α-l-rhamnopyranosyl-(1→2)-*O*-[α-l-rhamnopyranosyl-(1→6)]-β-d-glucopyranoside were isolated from the leaves of *Dracaena thalioides* Makoy ex E. Morris, a perennial plant native to tropical regions of Africa [78].

#### 2.1.9. Colorants of Dragon’s Blood Resins

**Figure 17:**
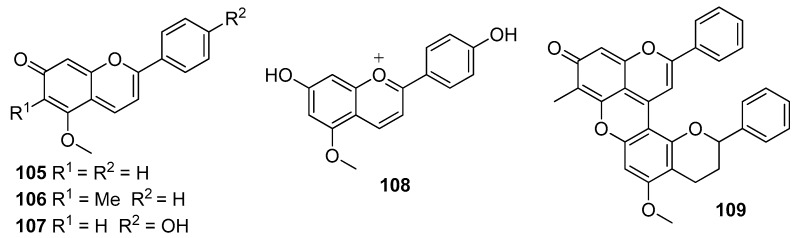
Chemical structures of compounds **105**–**109** responsible for the red color of dragon’s blood resin from *Dracaena* species.

Different red colorants, namely, dracaenin A (**79**) [42,54] (Figure 12), nordrachorhodin (**105**), dracorhodin (**106**), dracoflavilium (**107**/**108**), and dracorubin (**109**) (Figure 17), have been isolated from dragon’s blood resins obtained from different *Dracaena* trees [79]. The compounds share a common chromophore constituted by three conjugated rings forming a 2-phenyl-1-benzopyran moiety.

**Figure 18:**
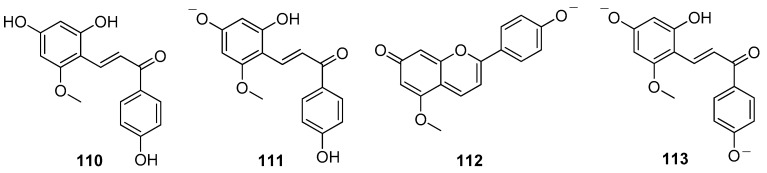
Species **110**–**113** in equilibrium with dracoflavilium (**107/108**) in aqueous solution at different pH.

These compounds undergo multiple structural transformations in aqueous acidic and basic solution, following the same basic mechanisms, that were completely characterized in the case of 7,4′-dihydroxy-5-methoxyflavilium, called dracoflavilium (**107/108**) [79]. This colorant was isolated from a resin extracted from *D. draco* centenary trees existing in the region of Lisbon and Madeira island; the structure was confirmed by total synthesis [79]. Dragon’s blood is yellow in strongly acidic aqueous solutions, because the species present is the flavylium cation **108**. At moderately acidic pH values, the red quinoid base **107** is formed which absorbs at about 550 nm. This base is the major species (63%) at biological pH (ca. 6) and gives the resin the characteristic red color (Figure 1a). The remaining species (37%) in the equilibrium is the pale-yellow (*E*)-chalcone **110**. Ionized (*E*)-chalcone **111** and the predominant pink deprotonated quinoid base **112** are present at pH = 8–9, whereas the double deprotonated base **113** is the only species occurring at pH = 12 (Figure 18) [79].

### 2.2. Sansevieria Species

#### 2.2.1. *Sansevieria cylindrica* Bojer ex Hook. (*Dracaena angolensis* (Welw. ex Carrière) Byng & Christenh)

**Figure 19:**
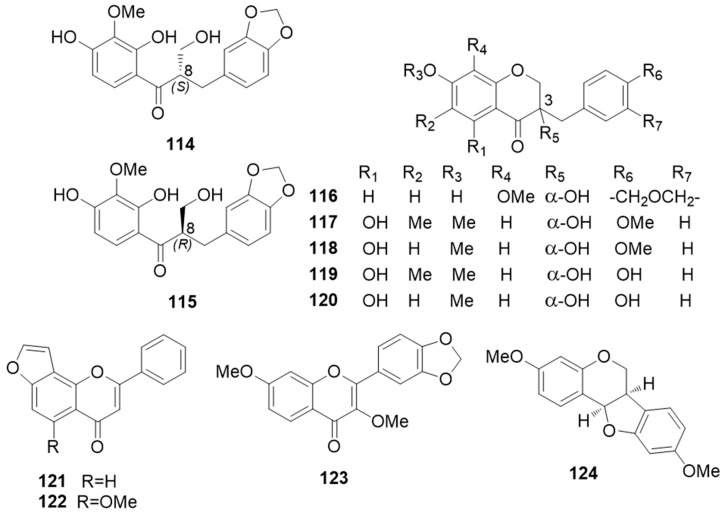
Chemical structures of compounds **114**–**124** isolated from *S. cylindrica.*

*Sansevieria cylindrica* (Figure 1c), known with the indigenous names of cylindrical snake plant, African spear, spear sansevieria, and Saint Bárbara sword in Brazil, is a succulent plant native to Angola. In addition to ornamental uses, the whole plant is used in Myanmar traditional medicine for treating cuts, sprains, and broken bones, whereas the roots are used to cure snakebites. The hydroxymethyldihydrochalcone (1,2*-seco*-homoisoflavanone) (+)-2′,4′-dihydroxy-3′-methoxy-3,4-methylenedioxy-8-hydroxymethyldihydrochalcone, named (+)-(8*S*)-trifasciatine C (**114**), was isolated from the aerial parts of *S. cylindrica* [80]. It showed moderate cytotoxic effects against human breast adenocarcinoma (MCF7) cells. From the aerial parts of *S. cylindrica* Said et al. also isolated 3,7-dihydroxy-8-methoxy-3-(3′,4′-methylenedioxybenzyl) chroman-4-one, named (+)-(3*S*)-trifasciatine B (**116** ≡ *ent*-**127**). This compound exhibited significant DPPH radical scavenging activity (IC_50_ 35.2 µg/mL), that was comparable with that of ascorbic acid (IC_50_ 33.3 µg/mL) [81]. Phytochemical investigations of *S. cylindrica* rhizomes, collected in Myanmar, revealed the presence of (-)-(8*R*)-trifasciatine C (**115** ≡ *ent*-**114**), homoisoflavanones (-)-(3*R*)-trifasciatine A (**126**), (+)-trifasciatine B (**116**), compounds **117**–**120** (Figure 19), and (-)-cambodianol (**20**) [30], along with the furanoflavones lanceolatin B (**121**) and pongaglabol methyl ether (**122**), the flavone de(s)methoxy-kanugin (**123**), and the pterocarpan (-)-(6a*R*, 11a*R*)-homopterocarpin (**124**) [82,83].

Compounds **115**, **116**–**120** displayed weak radical scavenging activity in the DPPH test (EC_50_ values > 100 mM) [82]. Lanceolatin B (**121**) showed high cancer chemopreventive potential [84], and anti-neuroinflammatory and analgesic properties [85].

#### 2.2.2. *Sansevieria roxburghiana* Schult. & Schult. f.

**Figure 20:**
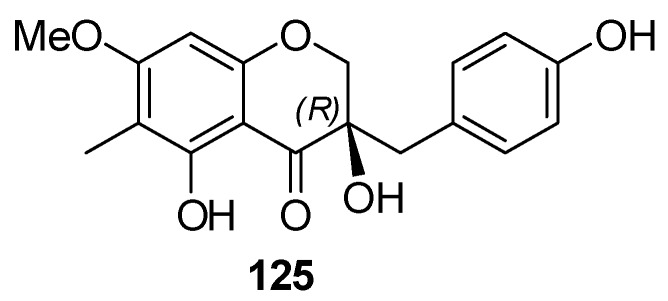
Chemical structure of compound **125** isolated from *S. roxburghiana.*

The homoisoflavanone (-)-(3*R*)-cambodianol (**20**) was claimed to have been isolated from a MeOH extract of *S. roxburghiana* [86]. However, recent careful examination of the NMR spectra of the compound led to a revision of the structure. In fact, the correct structure corresponds to that of compound **125** (Figure 20), that is the enantiomer of homoisoflavanone **119** isolated from *S. cylindrica* [82,83].

#### 2.2.3. *Sansevieria trifasciata* Prain (*Dracaena trifasciata* (Prain) Mabb)

**Figure 21:**
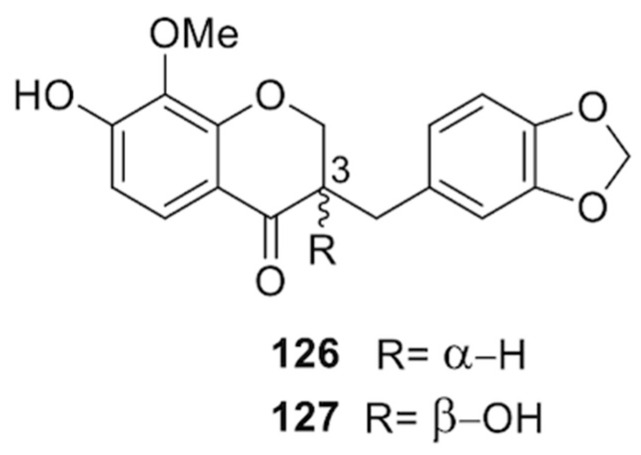
Chemical structures of compounds **126** and **127** isolated from *S. trifasciata.*

Tchegnitegni and collaborators isolated two sappanin-type homoisoflavonoids, named trifasciatine A (**126**) and (-)-(3*R*)-trifasciatine B (**127** ≡ *ent*-**116**) (Figure 21), from the EtOAc soluble fraction of a methanol extract of *S. trifasciata* [87]. Dihydrochalcone (+)-(8*S*)-trifasciatine C (**114**) was isolated from the aerial parts [88].

## 3. Chemical Structures and Chemotaxonomic Considerations

A wide variety of flavonoids and stilbenoids have been isolated from *Dracaena* species. They cover the families of flavones (e.g., **31**), flavans (e.g., **2**), flavanones (e.g., **12**), homoisoflavans (e.g., **22**), *meta*-homoisoflavans (e.g., **55**), homoisoflavones (e.g., **3**), homoisoflavanones (e.g., **13**), chalcones (e.g., **10**), dihydrochalcones (e.g., **7**), chalcanes (e.g., **34**), stilbenes (e.g., **43**), and flavonoid dimers and trimers. Dimeric structures include flavan-flavan (e.g., **15**), chalcane-flavan (e.g., **17**), chalcone-dihydrochalcone (e.g., **24**), chalcane-dihydro chalcone (e.g., **26**), chalcane-homoisoflavan (e.g., **29**), chalcane-stilbene (e.g., **60**), chalcane-flavilium (e.g., **79**), homoisoflavan-stilbene (e.g., **82**), dihydrochalcone-stilbene (e.g., **84**), and dihydrochalcone-homoisoflavan dimers (e.g., **86**). Other dimers include a stilbene-phenylpropanoid dimer (**44**) and flavan-phenylpropanoid dimers (e.g., **5**). Trimeric structures include chalcane-chalcane-flavan (e.g., **25**), chalcane-chalcane-chalcane (e.g., **30**), and dihydrochalcone-chalcane-chalcane trimers (e.g., **89**). Interestingly, in contrast to these flavonoids, *Dracaena* anthocyanidins and anthocyanidin have poorly been characterized. Moreover, it is worth noting that the absolute configurations of several flavans, homoisoflavans, homoisoflavanones, and flavonoid dimers and trimers have not been determined. Nonetheless, the stereochemistry should be assigned, both for completing the structural determination of isolated compounds and for a better understanding of compound bioactivities.

Among flavonoid families, dihydrochalcones, flavans, homoisoflavans, *meta*-homoisoflavans, and oligomers appear to be diagnostic chemical clusters in *Dracaena* species. Loureins A (**36**) and B (**37**) are often used as the chemical markers for the quality control of dragon’s blood.

In contrast to the wide variety of *Dracaena* flavonoids, the chemical families mostly occurring in *Sansevieria* species are only flavones (e.g., **121**) and homoisoflavanones (e.g., **116**). This finding may depend on the fact that, compared with *Dracaena* species, less phytochemical studies have been devoted to *Sansevieria* spp. Moreover, *Dracaena* flavonoids have mostly been isolated from red resins, while those identified in *Sansevieria* have been found in extracts of aerial parts and rhizomes. Interestingly, the pterocarpan **124** and the 8-hydroxymethyldihydrochalcones (1,2-*seco*-homoisoflavanones) **114** and **115**, isolated from two *Sansevieria* spp., are the only examples of such structures occurring in the two groups of plants. In addition to flavones, homoisoflavanones are the only flavonoids isolated from both *Sansevieria* and *Dracaena* species, although they display different substitution patterns. In fact, compared with *Dracaena* homoisoflavanones, the structures of the corresponding derivatives from *Sansevieria* spp. usually contain more *O*- and *C*-alkyl substituents; moreover, a hydroxy group is often bonded to C3. Enantiomeric trifasciatines B (**116** and **127**) and C (**114** and **115**), and homoisoflavanones **119** and **125** are examples of antipodal congeners occurring in different species of the same genus. More interesting is the isolation of enantiomeric compounds from different parts of the same plant. Thus, (+)-(8*S*)-trifasciatine C (**114**) was isolated from aerial parts of *S. cylindrica* [80], while the (8*R*)-enantiomer (**115**) was isolated from rhizomes [82]. Two different biosynthetic routes have been proposed to explain the formation of such antipodal flavonoids. Enantiodivergent biosynthetic reactions are involved that stem from achiral intermediates [82,87]. The isolation of homoisoflavonoids appears to have chemotaxonomic significance, because the largest number have been associated with Asparagaceae, Fabaceae and, to a minor extent, Liliaceae and Orchidaceae families [89].

## 4. Biological and Pharmacological Properties

The biological and pharmacological activities determined for dragon’s blood, crude and enriched extracts from *Dracaena* spp. are described in detail in reference [8]. These properties include analgesic, anti-inflammatory, antibacterial, hypolipidemic, hypoglycemic, and cytotoxic activities, as well as bidirectional regulation of hemorheology and cardiovascular and cerebrovascular effects. Pharmacological activities determined for individual flavonoids and stilbenoids isolated from *Dracaena* and *Sansevieria* species are summarized in Table 1. Standard in vitro biological assays have usually been carried out to measure the effects reported in the Table.

Especially remarkable are the anti-inflammatory activities of cochinchinenins L (**72**) and M (**73**) [56], and pterostilbene (**43**) [43], and the anti-neuroinflammatory properties of 4,4′-dihydroxy-2-methoxydihydrochalcone (**8**), flavones **31**–**33**, and homoisoflavan **59** [42]. The high cytotoxic effects exhibited by cambodianol (**20**) [30,31] and 4,4′-dihydroxy-3,2′-dimethoxychalcone [32] against different cell lines in an MTT assay are comparable with those the very well-known anti-cancer chemotherapy drugs paclitaxel and mitomycin C. The osteogenic effects of compounds **45**–**47**, **49**–**51** [49] and the thrombin-inhibiting activities of cochinchinenene A (**60**) and cochinchinenin C (**76**) [47] are highly interesting. The inhibitory activities of the pro-inflammatory enzymes COX-1 and COX-2 by compounds **100**–**102** [72], as well as the PTP1B inhibitory activities of biflavocochins B (**83**), F (**87**), and G (**88**) [53], the multifaceted activities of loureirin B (**94**) [69,72,73], and the high cancer chemopreventive potential, anti-neuroinflammatory, and analgesic properties of lanceolatin B (**121**) are also worthy of note [84,85].

It must be stressed that in vivo pharmacological studies of the activities and underlying mechanisms of dragon’s blood and extracts from *Dracaena* and *Sansevieria* species are still incomplete or had an inappropriate scientific approach. The results are thus scientifically invalid [8]. Therefore, although these natural remedies have attractive potential therapeutic effects on different diseases, especially for the treatment of cardiovascular and cerebrovascular diseases, there is an urgent need to conduct experiments on appropriate cell lines and animal models under scientifically correct protocols, before they can enter into clinical trials [8]. The same conclusion holds for individual flavonoids and stilbenes; namely, more scientific experiments, especially in vivo, must be performed before promising bioactive compounds can be promoted as approved effective drugs.

## 5. Conclusions

The field of natural product chemistry has greatly contributed to the progress of pharmacology and medicine. The wide range of bioactive compounds isolated from different plants still motivate many research groups worldwide to find new bioactive constituents and to determine their structures and biological activities. In this review we have reported the most characteristic structures and bioactivities of flavonoids and stilbenes isolated from *Dracaena* and *Sansevieria* species. These plants have revealed to be rich sources of compounds having unprecedented structures and exhibiting a wide range of biological and pharmacological properties. More attention has been devoted to *Dracaena* than *Sansevieria* flavonoids, possibly due to the very well-known effects of the red resin dragon’s blood, that is collected from some *Dracaena* species and is widely used in several Asian traditional medicines. Indeed, the number and diversity of flavonoids isolated from *Dracaena* dragon’s blood are impressive, encompassing a great number of chemical families. In addition, the potential therapeutic effects of the drug and individual constituents on different diseases are attractive, such as the remarkable activities on cardiovascular, inflammatory, and cerebrovascular diseases. However, more in vitro and in vivo scientific experiments must be programmed and carried out before the effective material may be qualified as a suitable candidate for clinical trials.

Compared to the phytochemical investigations conducted on *Dracaena* spp., those concerning *Sansevieria* species are less numerous and isolated flavonoids are chemically less diverse. However, the structural similarities of flavonoids isolated from *Sansevieria* and *Dracaena* plants seem to justify, on a chemotaxonomic basis, the recent inclusion of *Sansevieria* species inside the genus *Dracaena*.

## Figures and Tables

**Table 1:** Biological/pharmacological activities of characteristic flavonoids and stilbenoids isolated from *Dracaena* and *Sansevieria* species.

Source(s) and Compound(s)	Biological/pharmacological Activities	References
*Dracaena angustifolia*		
(2*R*)-3′,7-dihydroxy-5′,5-dimethoxy-8-methylflavone (**2**)	Anti-inflammatory activity: **2** (IC_50_ = 45 µM); **3** (IC_50_ = 33 µM); **4** (IC_50_ = 61 µM)	[23]
desmethylisoophiopogonone B (**3**)		
5,7,4′-trihydroxyhomoisoflavanone (**4**)		
*Dracaena cambodiana*		
cambodianins A (**5**) and B (**6**); 4,4′-dihydroxy-2′-methoxychalcone (**11**) (3*R*)-7,4′-dihydrohomoisoflavanone (**13**)	Cytotoxic effects against chronic myelogenous leukemia K-562 (**5**, IC_50_ = 13.300 ± 0.064 µg/mL; **6**, IC_50_ = 14.100 ± 0.042 µg/mL; **11**, IC_50_ = 32.500 ± 0.082 µg/mL; **13**, IC_50_ = 15.600 ± 0.040 µg/mL), human hepatocarcinoma SMMC-7721 (**6**, IC_50_ = 25.000 ± 0.025 µg/mL; **11**, IC_50_ = 12.500 ± 0.025 µg/mL; **13**, IC_50_ = 30.200 ± 0.022 µg/mL), and human gastric tumor SGC-7901 (**6**, IC_50_ = 68.000 ± 0.226 µg/mL; **11**, IC_50_ = 11.000 ± 0.018 µg/mL; **13**, IC_50_ = 20.500 ± 0.082 µg/mL) cell lines. Antibacterial activity against *Staphylococcus aureus* and methicillin-resistant *S. aureus* (MRSA)	[24]
(±)-7,4′-dihydroxyhomoisoflavanone	Moderate cytotoxic effects against human myeloid leukemia (K562) and human gastric tumor (SGC-7901) cell lines (IC_50_ = 16–29 μg/mL)	[31]
4,4′-dihydroxy-2,3′-dimethoxydihydrochalcone (**7**)	Antibacterial activity against *S. aureus* and MRSA	[24,25]
4,4′-dihydroxy-2-methoxydihydrochalcone (**8**)		
cambodianins D (**14**) and E (**15**)		
(2*S*)-7,4′-dihydroxy-6,8-dimethylflavan (**16**)		
cambodianin G (**18**)	Cytotoxic effects against K-562 (IC_50_ = 9.5 µg/mL) and SGC-7901 (IC_50_ = 16.2 µg/mL) cell lines; antibacterial activity against *S. aureus*	[26]
cambodianin H (**19**)	Antibacterial activity against *S. aureus*	
cambodianol (**20**)	Cytotoxic effects against K562 (IC_50_ = 1.4 µg/mL), SGC-7901 (IC_50_ = 2.9 µg/mL) and SMMC-7721 (IC_50_ = 5.0 µg/mL) cell lines, comparable with the values of paclitaxel	[30,31]
8-methylsocotrin-4′-ol	Moderate cytotoxic effects against K562 and SGC-7901 cell lines	[31]
4,4′-dihydroxy-3,2′-dimethoxychalcone	Cytotoxic effects against K562, SMMC-7721, and SGC-7901 cell lines, with IC_50_ values of 2.5, 4.3, and 4.4 mg/mL, respectively, comparable with the values of mitomycin C	[32]
(2*R*)-7,4′-dihydroxy-6-methylflavan (**21**)	Weak cytotoxic effects (IC_50_ = 39.22 μM) against human hepatocellular carcinoma BEL-7402 cell line	[34]
(3*R*)-7,3′,4′-trihydroxyhomoisoflavan (**22**)	Acetylcholinesterase (AChE) inhibitory activity	[34]
*Dracaena cinnabari*		
4′-hydroxy-7,8-methylenedioxyhomoisoflavan (**23**)	Inhibition of NO (nitric oxide), TNF-α, and IL-6 production in lipopolysaccharide stimulated mouse macrophage RAW 264.7 cells	[36]
dracidione (**24**)	α-Glucosidase inhibitory activity (IC_50_ = 40.27 µg/mL)	[37]
*Dracaena cochinchinensis*		
4,4′-dihydroxy-2-methoxydihydrochalcone (**8**)	Anti-neuroinflammatory effects (IC_50_ = 10.29 ± 1.05 µM)	[42]
apigenin	Remarkable anti-neuroinflammatory activity (inhibition of NO production): apigenin (IC_50_ = 3.12 ± 1.27 µM); 7-hydroxy-3-(4-hydroxybenzylidene) chroman-4-one (IC_50_ = 4.46 ± 0.95 μM); **31** (IC_50_ = 4.31 ± 1.79 µM); **32** (IC_50_ = 8.00 ± 2.04 µM); **33** (IC_50_ = 3.36 ± 1.67 µM)	[42]
7-hydroxy-3-(4-hydroxybenzylidene)chroman-4-one		
7,3′-dihydroxy-4′-methoxyflavone (**31**)		
7,4′-dihydroxyflavone (**32**)		
7-hydroxyflavone (**33**)		
4′-hydroxy-2,4-dimethoxydihydrochalcone (**36**)	Anti-inflammatory activity	[43]
4′-hydroxy-2,4,6-trimethoxydihydrochalcone (**37**)		
(3*S*)-3,7,4′-trihydroxy-5-methoxyhomoisoflavanone (**41**)	Inhibitory activity against nitric oxide (NO) production: **41** (IC_50_ = 75.6 ± 1.2 µM); **42** (IC_50_ = 60.4 µM)	[52]
7,4′-dihydroxyhomoisoflavanone (**42**)		
pterostilbene (**43**)	NQO1 [NAD(P)H Quinone Dehydrogenase 1] inducing activity and anti-inflammatory effects	[43]
7,4′-dihydroxy-8-methylflavan (**45**)	Osteogenic effects on mesenchymal stem cells (MSCs)	[49]
5,4′-dihydroxy-7-methoxy-6-methylflavan (**46**)	Osteogenic effects on mesenchymal stem cells (MSCs) and anti-inflammatory activity	[43,49]
7,4′-dihydroxyflavan (**47**)	Osteogenic effects on mesenchymal stem cells (MSCs)	[49]
(2*S*)-7,4′-dihydroxy-8-methylflavan (**48**)	Antibacterial activities against *Helicobacter pylori* (MIC = 31.3 µM)	[47]
dracaeconolide B (**49**)	Osteogenic effects on mesenchymal stem cells (MSCs)	[49]
(3*R*)-7,4′-dihydroxy-8-methoxyhomoisoflavan (**50**)		
(3*R*)-7,4′-dihydroxyhomoisoflavan (**51**)		
(3*R*)-7,4′-dihydroxy-5-methoxyhomoisoflavan (**52**)	Anti-neuroinflammatory activity (IC_50_ = 8.50 ± 1.28 µM)	[42]
7,4′-dihydroxyhomoisoflavan (**53**)	Anti-inflammatory activity	[43]
6,4′-dihydroxy-7-methoxyhomoisoflavan (**54**)		
10-hydroxy-11-methoxydracaenone (**58**)	Inhibitory activity against NO production (IC_50_ = 62.4 ± 3.5 µM) and NQO1 inducing activity	[43,52]
cochinchinenene F (**61**)	NQO1 inducing activity	[43]
cochinchinenenes A (**60**), B (**63**), C (**64**), and D (**65**)	Thrombin inhibitory activity: **60** (IC_50_ > 9.5 µM); **63** (IC_50_ = 17.8 µM); **64** (IC_50_ = 26.7 µM); **65** (IC_50_ > 41.3 µM)	[47]
cochinchinenene E (**66**)	NQO1 inducing activity	[43]
“cochinchinenene G” (**68**)	Inhibitory activity against NO production (IC_50_ = 2.18 ± 1.43 µM)	[52]
(2*R*)-8-methylsocotrin-4′-ol (**69**)	Thrombin inhibitory activity (IC_50_ = 21.5 µM)	[47]
cochinchinenins L (**72**) and M (**73**)	Inhibitory effects on NO production in lipopolysaccharide (LPS)-stimulated BV-2 microglial cells: 72 (IC_50_ = 4.9 ± 0.4 µM); 73 (IC_50_ = 5.4 ± 0.6 µM)	[56]
cochinchinenin C (**76**)	Thrombin inhibitory activity (IC_50_ > 9.2 µM); antibacterial activity against *Helicobacter pylori* (MIC = 29.5 µM)	[47]
1-[5-(2-methoxy-4,4′- dihydroxydihydrochalconyl)]-1-(4-hydroxyphenyl)-3-(2-methoxy-4-hydroxyphenyl)propane	Thrombin inhibitory activity (IC_50_ = 26.3 µM)	[47]
biflavocochin A (**82**)	Neuroprotective effect on serum deficiency-induced cellular damage in neuroendocrine PC12 cells (pheochromocytoma cells derived from the adrenal gland of *Rattus norvegicus*)	[53]
biflavocochins B (**83**), F (**87**), and G (**88**)	Protein-tyrosine phosphatase 1B (PTP1B) inhibitory activity	[53]
(7*E*)-2,4-dihydroxy-1-methylstilbene (**91**)	Antioxidant activity and antifungal activity against *Exserohilum turcicum*, *Bipolaris maydis*, *Curvularia lunata*, and *Fusarium graminearum*	[58]
*Dracaena loureiri*		
loureirin B (**94**)	Modulatory activity on the TTX-R sodium channel in dorsal root ganglion (DRG) neurons; effect on insulin secretion of pancreatic β-cells, increase of the mRNA level of *Pdx*-1, *MafA*, and intracellular ATP level; suppression of inflammatory cytokines and inhibition of apoptosis through regulation of IL-6/STAT3/NF-κB signalling pathway	[70,73,74]
loureirin D (**96**)	Inhibition of NO production stimulated by LPS-activated RAW 264.7 murine macrophages (IC_50_ = 50.3 µM)	[48,70]
4,4′-dihydroxy-2,6-dimethoxydihydrochalcone (**97**) (also isolated from *D. cambodiana*)	Cytotoxic effects against K-562 (IC_50_ = 12.800 ± 0.015 µg/mL), SMMC-7721 (IC_50_ = 16.200 ± 0.040 µg/mL), and SGC-7901 (IC_50_ = 10.000 ± 0.060 µg/mL) cell lines; antibacterial activities against *S. aureus*; stimulation of the hormone-dependent MCF-7 cell growth in a concentration dependent manner between 10^−8^ and 10^−5^ M	[24,71]
4′,2-dihydroxy-4,6-dimethoxydihydrochalcone (**98**)	Stimulation of the MCF-7 cell proliferation in a concentration dependent manner between 10^−8^ and 10^−5^ M	[71]
4,3′,5′-trihydroxystilbene (**100**)	Inhibitory activities of the enzymes COX-1 and COX-2: **100** (IC_50_ = 2.61 and 2.16 µM, respectively); **101** (IC_50_ = 4.92 and 2.21 µM, respectively); **102** (IC_50_ = 4.84 and 1.29 µM, respectively)	[72]
4,3′-dihydroxy-5′-methoxystilbene (**101**)		
4-hydroxy-3′,5′-dimethoxystilbene (**102**)		
5,7,4′-trihydroxyhomoisoflavanone (**4**)	Binding affinity for the bovine uterine estrogen receptor (IC_50_ = 375 nM)	[71]
*Dracaena usambarensis*		
(3*S*)-3,4ʹ,5,6-tetrahydroxy-7-methoxyhomoisoflavanone (**104**)	Moderate cytotoxicity against drug sensitive human lymphoblastic leukemia CCRF-CEM cells	[76]
*Sansevieria cylindrica*		
(+)-trifasciatine C (**114**)	Moderate cytotoxicity against MCF7 cells (IC_50_ = 34.1 µg/mL)	[80]
(+)-trifasciatine B (**116**)	Weak DPPH radical scavenging activity (IC_50_ = 35.2 µg/mL)	[81]
lanceolatin B (**121**)	High cancer chemopreventive potential, anti-neuroinflammatory and analgesic properties	[84,85]
